# The relationship between time of admission and icu outcome

**DOI:** 10.1186/2197-425X-3-S1-A529

**Published:** 2015-10-01

**Authors:** G Warnock

**Affiliations:** Intensive Care Department, Victoria Infirmary/NHS Greater Glasgow & Clyde, Glasgow, United Kingdom

## Introduction

The relationship between admission time and ICU outcome is contentious with studies showing it is associated with a poor outcome [1], and contradictory studies that suggest there is no difference [2]. This study compared ICU outcomes between daytime (DT) and night-time (NT) admissions in our local medical intensive care unit. NT admissions were defined as those between 20:00 and 07:59.

## Objectives

To assess if there was any difference in outcome between daytime and night-time admissions in our local intensive care unit.

## Methods

A retrospective data search was carried out utilising WardWatcher^TM^ data over the period of 01/01/2014 to 31/12/2014. A total of 243 patients were admitted over this period; 26 were excluded due to incomplete APACHE-II data.

## Results

A total of 107 patients were admitted during daytime hours vs. 110 during night-time hours. There was no significant difference between the mean age of the patients (58.9 vs 59). The majority of patients were admitted from General Medical or General Surgical specialties (DT admissions 60.7% (n = 65), and 33.6% (n = 36); NT admissions 60.9% (n = 67), and 31.8% (n = 35)). There was an increased actual mortality rate (AMR) between NT and DT admissions (AMR 17.3% (n = 19) vs. 25% (n = 27)), but no significant difference in standardised mortality ratio (SMR) - 0.59 vs. 0.40. There was also an increased length of stay associated with NT admissions (6.6 days vs. 4.8 days).

## Conclusions

Our study showed there was an increased AMR in DT vs. NT admissions but no significant difference in SMR. There was an increased length of stay associated with night-time admissions which has implications for the cost of patient care.
